# Survival Nomogram for Lung Adenocarcinoma Patients With Bone Metastasis Based on the SEER Database and an External Validation Cohort

**DOI:** 10.1002/cnr2.70211

**Published:** 2025-05-05

**Authors:** Zhiming Liu, Min Zhang, Shuo Han, Hao Zhang, Shengwei Meng, Zhubin Shen, Xuexiao Ma

**Affiliations:** ^1^ Department of Spine Surgery The Affiliated Hospital of Qingdao University Qingdao China; ^2^ Department of Neonatology The Second Affiliated Hospital and Yuying Children's Hospital of Wenzhou Medical University Wenzhou China; ^3^ Department of Spine Surgery China‐Japan Union Hospital of Jilin University Changchun China

**Keywords:** bone metastasis, chemotherapy, lung adenocarcinoma, overall survival, radiotherapy, survival nomogram

## Abstract

**Background:**

Lung adenocarcinoma is a common type of cancer that can lead to bone metastasis and has a poor prognosis. Although previous studies have established nomograms for lung adenocarcinoma, these nomograms do not effectively predict the prognosis of lung adenocarcinoma patients with bone metastasis. This study aims to establish and validate a new nomogram to solve this problem.

**Methods:**

Data were collected from the SEER database and from patients at our hospital who had been diagnosed with lung adenocarcinoma and developed bone metastases. The patients were randomly assigned into the training and internal validation sets in a 7:3 ratio. External validation was conducted using an independent patient cohort from two hospitals. Different methods were used to evaluate the nomogram's performance. The relationship between different metastatic sites and radiotherapy and chemotherapy was also analyzed to evaluate patient prognosis.

**Results:**

The following factors were identified as significant prognostic indicators: age, sex, marital status, T stage, N stage, tumor grade, tumor size, presence of brain and liver metastases, and receipt of chemotherapy. The nomogram's concordance indices for predicting overall survival were consistently above 0.7, and the area under the curve values, calibration plots, and decision curves all confirmed the nomogram's strong predictive accuracy. Moreover, our analysis revealed that chemotherapy was the most effective treatment modality.

**Conclusions:**

This study developed a nomogram that can predict the prognosis of lung adenocarcinoma patients with bone metastasis. The results showed that patients with liver metastasis had the worst prognosis and that chemotherapy was the most effective treatment regimen for patients with different metastatic sites.

## 1 Introduction

Lung adenocarcinoma is the leading cause of cancer‐related deaths worldwide [1]. In lung cancer, bone metastasis is frequent, occurring in approximately 30%–40% of cases [2]. Bone metastases often lead to bone pain, pathological fractures, spinal cord compression, and hypercalcemia, which can severely affect patients' quality of life and are the primary causes of mortality [3, 4]. In the 8th edition of the TNM staging system, the number of metastatic sites was considered as the basis for staging, and it was divided into M1b and M1c. However, the TNM staging system does not consider the patient's overall health status, disease characteristics, and treatment approach, making it difficult to accurately predict the prognosis of patients with metastatic lung cancer [5]. Hence, novel prediction models to assess the overall survival (OS) of patients with lung adenocarcinoma and bone metastasis need to be developed.

The nomogram is widely used for predicting the prognosis of tumors. It is user‐friendly, and the results can be quickly obtained with high accuracy, which is very helpful for clinical decision‐making [6]. Although nomograms have been widely used in previous studies to estimate the OS of patients with lung adenocarcinoma [7, 8, 9], they have not been used to predict the prognosis of lung adenocarcinoma patients with bone metastasis. Therefore, the present study aimed to develop a novel nomogram to enhance the accuracy of predicting the prognosis of patients diagnosed with lung adenocarcinoma and bone metastasis. Moreover, the tumor metastasis site and the treatment method (radiotherapy and chemotherapy) in patients were comprehensively analyzed to investigate the effect of the treatment method.

This manuscript is written in accordance with the TRIPOD checklist [10].

## 2 Methods

### 2.1 Data Source and Patient Selection

All patient data in this study were obtained from the SEER database (www.seer.cancer.gov) and downloaded via the SEER Stat software (version 8.4.0.1). The data were updated in April 2022, and based on submissions in November 2021, the target population was 17 registries in the United States for the period of 2010–2019. During data collection, patient IDs were used to collect information from individual participants.

### 2.2 Inclusion and Exclusion Criteria

The inclusion criteria were as follows: patients with a pathological diagnosis of lung adenocarcinoma, patients with bone metastasis, patients with complete clinical and pathological data, and patients aged over 18 years at diagnosis. The exclusion criteria were as follows: patients with unknown marital status, T stage, N stage, tumor grade, or distant metastasis site; diagnostic method not based on pathology; and presence of primary tumors in other locations. All selected patients were randomly assigned to a training set and a validation set in a 7:3 ratio.

**FIGURE 1:**
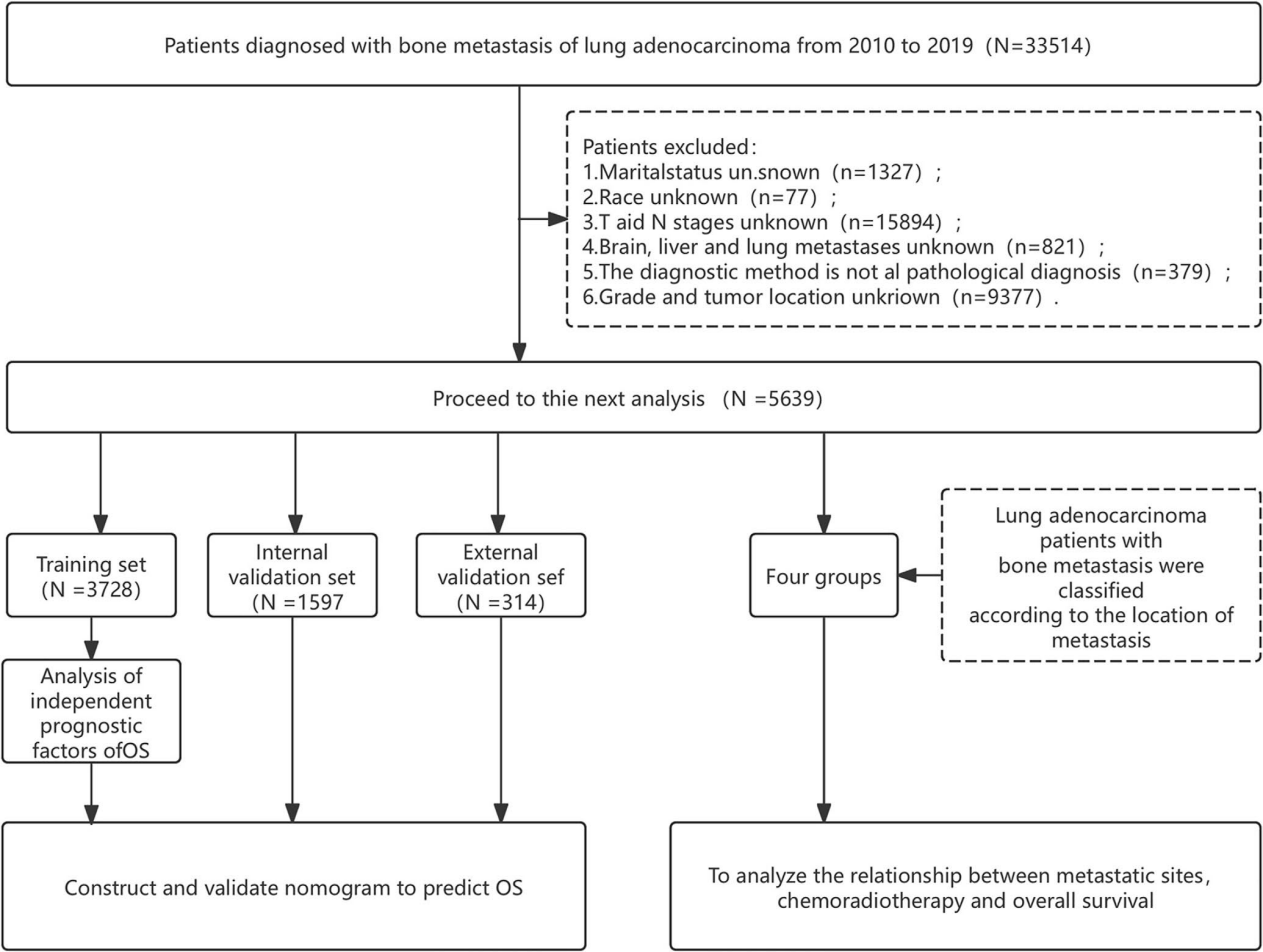
Flowchart of patient screening. *N* = total number of patients at each stage.

To validate the nomogram developed in this study, an external validation cohort was included. This cohort comprised 314 patients diagnosed with lung adenocarcinoma and bone metastasis between May 2019 and June 2023 at Qingdao University Affiliated Hospital and Qingdao Municipal Hospital. The same inclusion and exclusion criteria that were previously applied were used. Ethical approval for this study was obtained from the Ethics Committee of the Affiliated Hospital of Qingdao University, and the specific screening flowchart is presented in Figure 1.

### 2.3 Variables

The demographic and clinicopathological data of patients included age at diagnosis, marital status, T stage, N stage, tumor grade, tumor size and location, and distant metastasis sites. The X‐Tile software (version 3.6.1) was used to analyze the optimal cutoff values of age and tumor size and to convert them into categorical data. The cutoff values for age were 65 and 72 years, and the cutoff values for tumor size were 41 and 64 mm, which were divided into three subgroups. OS was defined as the time from diagnosis to death (whatever the cause). Furthermore, the patients included in this study were categorized into four subgroups based on the tumor metastasis sites (i.e., bone metastasis alone, bone and lung metastasis, bone and brain metastasis, and bone and liver metastasis). Subsequently, the impact of chemotherapy, radiotherapy, or a combination of both on the prognosis of patients in these four subgroups was investigated.

The mutation rates of epidermal growth factor receptor (EGFR) and anaplastic lymphoma kinase (ALK) in lung adenocarcinoma are very low [11, 12]. Moreover, the area under the curve (AUC) values of their receiver operating characteristic (ROC) curves are significantly lower than those in our nomogram. Therefore, biomarkers such as EGFR and ALK were not included in the nomogram model to enhance the model's universality.

### 2.4 Statistical Analysis

In this study, all data analyses were performed using R software (version 4.2.1). The Chi‐Square test was used to assess differences between the training and validation datasets. Univariate Cox regression analysis was used to evaluate the demographic and clinicopathologic factors, and factors with a *p*‐value less than 0.05 were further analyzed using multivariate Cox regression analysis. After multivariate Cox regression analysis, factors with a *p*‐value less than 0.05 were designated as independent prognostic factors. Moreover, the total score for each patient was computed, and all patients were categorized into high‐risk and low‐risk groups using the X‐Tile software (version 3.6.1).

### 2.5 Construction and Validation of a Nomogram

A nomogram was developed using the “rms” (version 6.3‐0) R package in R software (version 4.2.1) to predict the OS of patients at 6, 12, and 18 months. Then, ROC curves were plotted using the “pROC” R package (version 1.18.0), and the AUC values were calculated. Calibration curves were generated using the “survival” R package (version 3.4‐0) to assess the nomogram's accuracy, and decision curve analysis was performed using the “ggDCA” R package (version 1.2) to evaluate the nomogram's clinical utility. The optimal threshold for the total score was determined using the X‐Tile software (version 3.6.1), and all patients were divided into high‐risk and low‐risk subgroups accordingly. Kaplan–Meier (KM) survival curves were plotted using the “survminer” R package (version 0.4.9), and the log‐rank test was used to compare OS differences between subgroups.

### 2.6 Establishment of a Risk Classification System

The risk score for each patient with lung adenocarcinoma was calculated based on the established nomogram, and the optimal cutoff value (192) for all patients was determined using the X‐Tile software (version 3.6.1). Patients were categorized into high‐risk (≥ 192) and low‐risk (< 192) subgroups and visualized through KM curves.

### 2.7 Establishment of KM Curves to Determine the Therapeutic Effects at Different Distant Metastasis Sites

All patients were divided into four subgroups on the basis of the metastasis sites: bone metastasis alone, bone and brain metastases, bone and liver metastases, and bone and lung metastases. To study the therapeutic effects of different treatment methods on these four subgroups, KM curves were used to evaluate the effects of radiotherapy, chemotherapy, and combined treatment. The log‐rank method was used to calculate the *p*‐value, hazard ratio (HR), and 95% confidence intervals (CIs). Subsequently, the “survminer” R package (version 0.4.9) was used to visualize and generate the KM curves.

## 3 Results

### 3.1 Basic Characteristics of Patients

A total of 5325 patients with lung adenocarcinoma were included in this study. They were randomly allocated to a training set (3728 patients) and a validation set (1597 patients) in a 7:3 ratio. In addition, 314 lung adenocarcinoma patients with bone metastasis at two other hospitals served as an external validation set. The basic information and clinicopathological data of patients in the training set are shown in Table 1. The majority of patients were married (2192, 58.8%), men (1969, 52.8%), and under 65 years of age (1591, 42.7%) and had T4 (1897, 51.0%), N2 (1759, 47.2%), and grade III tumors (2193, 58.8%). Most patients had tumors located in the right (2173, 58.3%) and upper (2334, 62.6%) lung and had a tumor size of < 47 mm (1509, 40.5%). A total of 2347 patients (63.0%) received chemotherapy, and 2005 patients (53.8%) received radiotherapy.

**TABLE 1:** Demographic and clinicopathological characteristics of the training set and validation set.

	Training cohort (*n* = 3728)		Internal validation cohort (*n* = 1597)		External validation cohort (*n* = 314)		*p*
Age (years)							0.305
< 65	1591	42.7%	716	44.8%	91	29.0%	
65–72	857	23.0%	347	21.7%	85	27.1%	
> 72	1280	34.3%	534	33.5%	138	43.9%	
Sex							0.115
Female	1759	47.2%	714	44.7%	147	46.6%	
Male	1969	52.8%	883	55.3%	167	53.4%	
Marital status							0.258
Married	2192	58.8%	960	60.1%	238	76.1%	
Unmarried	1536	41.2%	637	39.9%	76	23.9%	
T stage							0.689
*T* ≤ 1	449	12.0%	205	12.8%	42	13.5%	
T2	1161	31.1%	494	30.9%	64	20.3%	
T3	221	5.9%	102	6.4%	19	6.1%	
T4	1897	51.0%	796	49.9%	189	60.1%	
N stage							0.268
N0	815	21.9%	356	22.3%	80	25.4%	
N1	346	9.3%	122	7.6%	27	8.6%	
N2	1759	47.2%	782	49.0%	149	47.5%	
N3	808	21.6%	337	21.1%	58	18.5%	
Grade							0.769
I	252	6.8%	98	6.1%	33	10.5%	
II	1237	33.2%	526	32.9%	86	27.4%	
III	2193	58.8%	955	59.8%	190	60.5%	
IV	46	1.2%	18	1.2%	5	1.6%	
Brain metastasis							0.258
No	2784	74.7%	1172	73.4%	235	74.9%	
Yes	944	25.3%	425	26.6%	79	25.1%	
Liver metastasis							0.636
No	2984	80.0%	1268	79.4%	245	78.0%	
Yes	744	20.0%	329	20.6%	69	22.0%	
Tumor size (mm)							0.756
< 47	1509	40.5%	666	41.7%	127	40.5%	
47–77	1060	28.4%	438	27.4%	78	24.8%	
> 77	1159	31.1%	493	30.9%	109	34.7%	
Radiotherapy							0.563
Yes	2005	53.8%	864	54.1%	152	48.4%	
No	1723	46.2%	733	45.9%	162	51.6%	
Chemotherapy							0.322
Yes	2347	63.0%	969	60.7%	180	57.3%	
No	1381	37.0%	628	39.3%	134	42.7%	
Tumor site							0.356
Upper lobe	2334	62.6%	1010	63.2%	180	57.3%	
Middle lobe	206	5.5%	75	4.7%	48	15.3%	
Lower lobe	1188	31.9%	512	32.1%	86	27.4%	
Laterality							0.805
Left	1555	41.7%	675	42.3%	128	40.8%	
Right	2173	58.3%	922	57.7%	186	59.2%	

### 3.2 Univariate and Multivariate Cox Regression Analyses of Lung Adenocarcinoma Patients With Bone Metastasis

**FIGURE 2:**
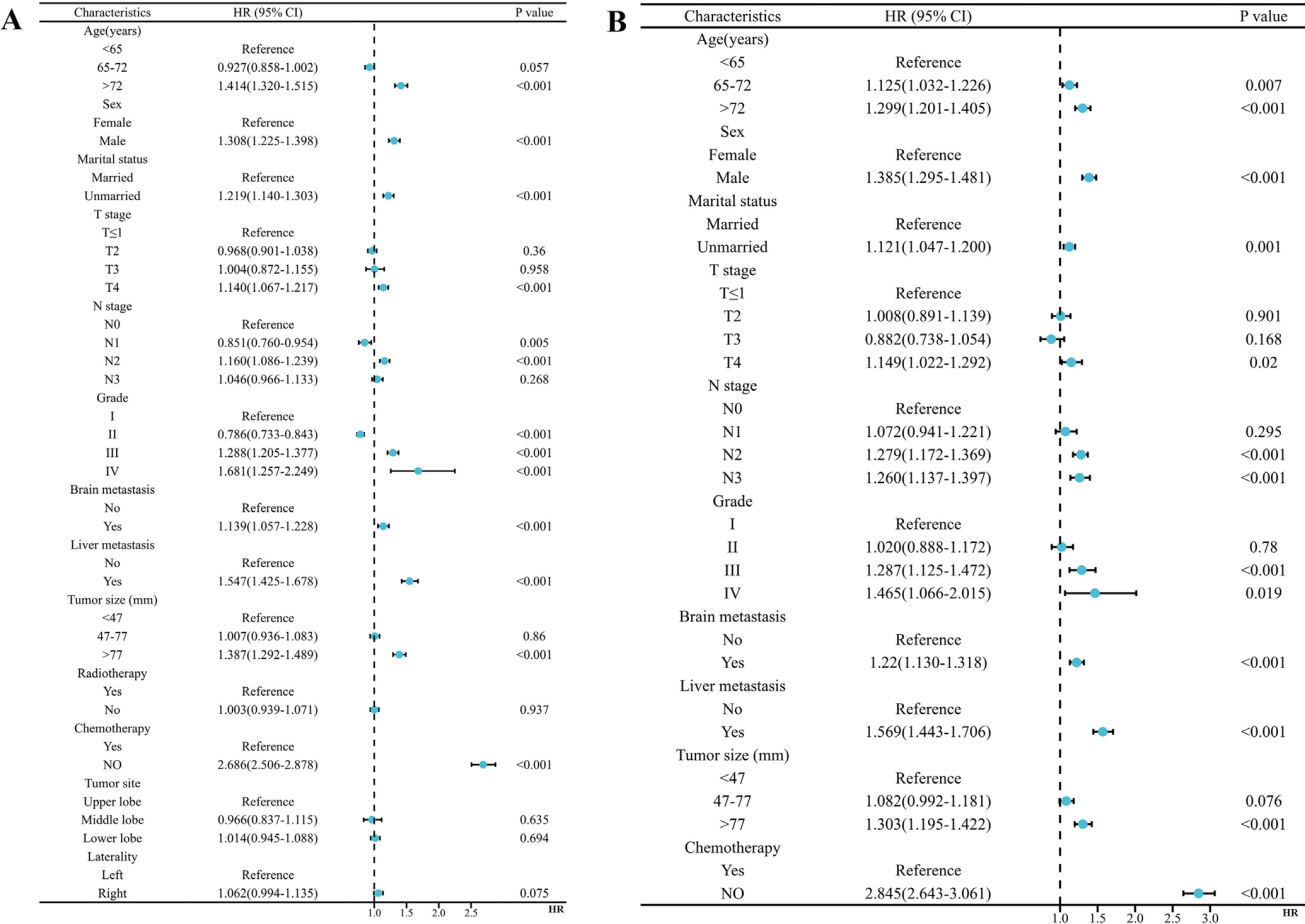
(A) Univariate and (B) multivariate Cox regression analyses and forest plot of patients with lung adenocarcinoma and bone metastasis. “Hazard Ratio (HR)” for the *X*‐axis and “Variables” for the *Y*‐axis. *p*‐Values < 0.05 were considered statistically significant.

The results of univariate and multivariate Cox regression analyses are presented in Table 2. In addition, a forest plot visualizing the results is shown in Figure 2. Univariate Cox regression analysis identified factors with a *p*‐value of less than 0.05 as significant, including age, gender, marital status, tumor size, T stage, N stage, tumor grade, presence of brain and liver metastases, and receipt of chemotherapy. Multivariate Cox regression analysis using these factors showed that the *p*‐values of these factors were all less than 0.05, indicating that these factors were independent prognostic factors for lung adenocarcinoma with bone metastasis. All independent prognostic factors were associated with a high risk of death; however, patients who received chemotherapy had a low risk of death.

**TABLE 2:** Univariate and multivariate cox regression analysis of lung adenocarcinoma patients with bone metastasis.

Variables	Univariate analysis			Multivariate analysis		
	HR	95% CI	*p*	HR	95% CI	*p*
Age (years)						
< 65	Ref.	—	—	Ref.	—	—
65–72	0.927	0.858–1.002	0.057	1.125	1.032–1.226	0.007
> 72	1.414	1.320–1.515	< 0.001	1.299	1.201–1.405	< 0.001
Sex						
Female	Ref.	—	—	Ref.	—	—
Male	1.308	1.225–1.398	< 0.001	1.385	1.295–1.481	< 0.001
Marital status						
Married	Ref.	—	—	Ref.	—	—
Unmarried	1.219	1.140–1.303	< 0.001	1.121	1.047–1.200	0.001
T stage						
*T* ≤ 1	Ref.	—	—	Ref.	—	—
T2	0.968	0.901–1.038	0.360	1.008	0.891–1.139	0.901
T3	1.004	0.872–1.155	0.958	0.882	0.738–1.054	0.168
T4	1.140	1.067–1.217	< 0.001	1.149	1.022–1.292	0.020
N stage						
N0	Ref.	—	—	Ref.	—	—
N1	0.851	0.760–0.954	0.005	1.072	0.941–1.221	0.295
N2	1.160	1.086–1.239	< 0.001	1.279	1.172–1.369	< 0.001
N3	1.046	0.966–1.133	0.268	1.260	1.137–1.397	< 0.001
Grade						
I	Ref.	—	—	Ref.	—	—
II	0.786	0.733–0.843	< 0.001	1.020	0.888–1.172	0.780
III	1.288	1.205–1.377	< 0.001	1.287	1.125–1.472	< 0.001
IV	1.681	1.257–2.249	< 0.001	1.465	1.066–2.015	0.019
Brain metastasis						
No	Ref.	—	—	Ref.	—	—
Yes	1.139	1.057–1.228	< 0.001	1.220	1.130–1.318	< 0.001
Liver metastasis						
No	Ref.	—	—	Ref.	—	—
Yes	1.547	1.425–1.678	< 0.001	1.569	1.443–1.706	< 0.001
Tumor size (mm)						
< 47	Ref.	—	—	Ref.	—	—
47–77	1.007	0.936–1.083	0.860	1.082	0.992–1.181	0.076
> 77	1.387	1.292–1.489	< 0.001	1.303	1.195–1.422	< 0.001
Radiotherapy						
Yes	Ref.	—	—			
No	1.003	0.939–1.071	0.937			
Chemotherapy						
Yes	Ref.	—	—	Ref.	—	—
No	2.686	2.506–2.878	< 0.001	2.845	2.643–3.061	< 0.001
Tumor site						
Upper lobe	Ref.	—	—			
Middle lobe	0.966	0.837–1.115	0.635			
Lower lobe	1.014	0.945–1.088	0.694			
Laterality						
Left	Ref.	—	—			
Right	1.062	0.994–1.135	0.075			

### 3.3 Establishment and Validation of a Nomogram

**FIGURE 3:**
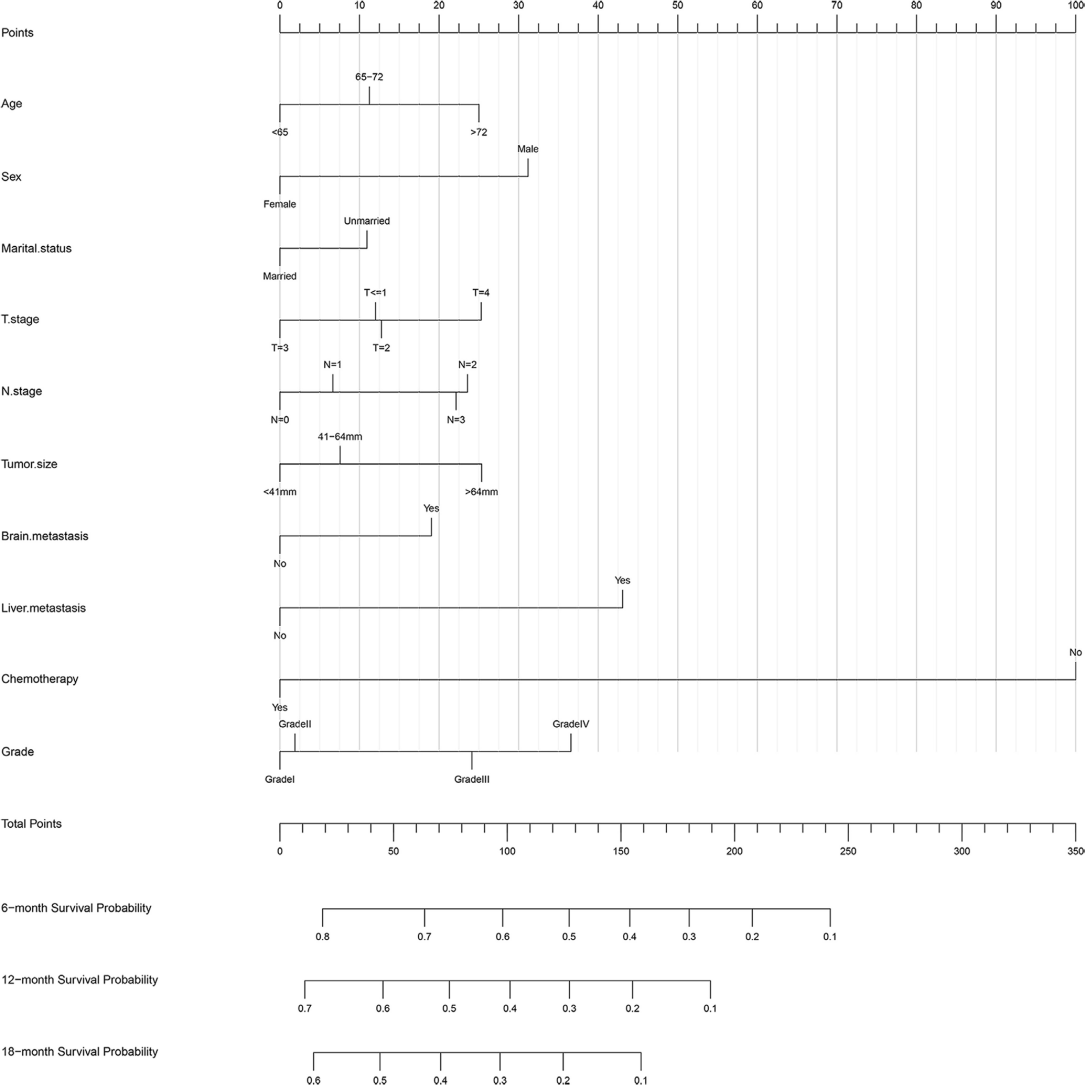
Nomogram predicting overall survival of patients at 6, 12, and 18 months. Instructions for using a nomogram (draw a vertical line to the points axis for each variable, sum the points, and locate the total on the survival probability axis).

**FIGURE 4:**
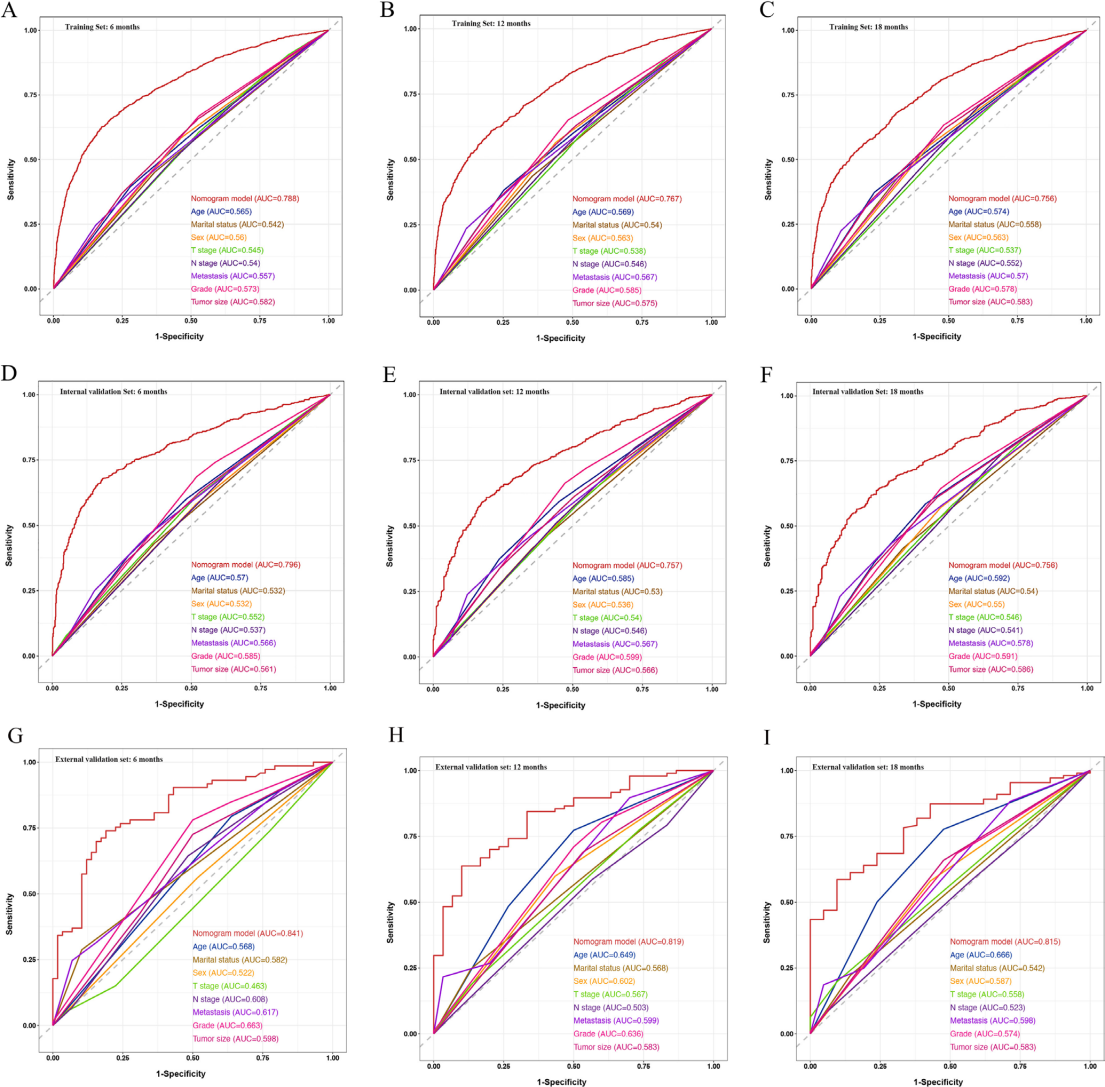
Receiver operating characteristic (ROC) curves of the nomogram and other independent risk factors at 6, 12, and 18 months. Training set (A, B, C); internal validation set (D, E, F); external validation set (G, H, I).

**FIGURE 5:**
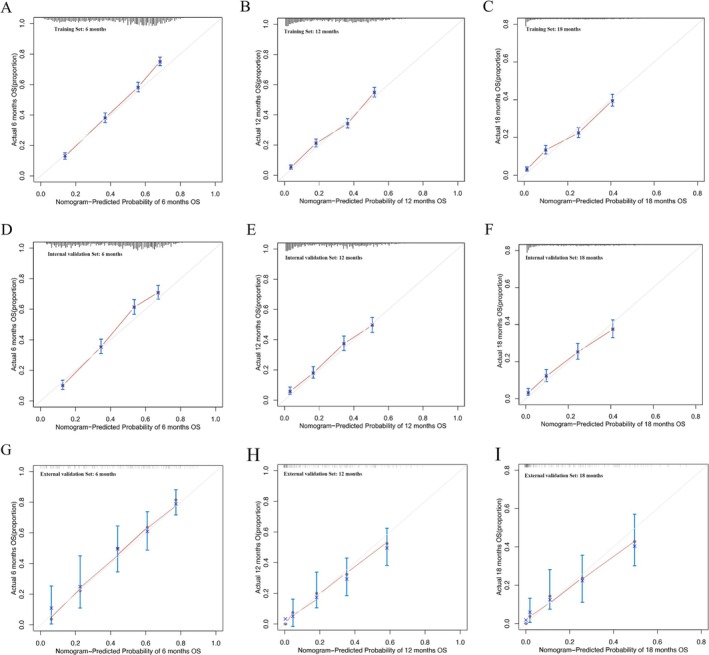
Calibration curves for predicting the overall survival (OS) of patients at 6, 12, and 18 months. Training set (A, B, C); internal validation set (D, E, F); external validation set (G, H, I).

**FIGURE 6:**
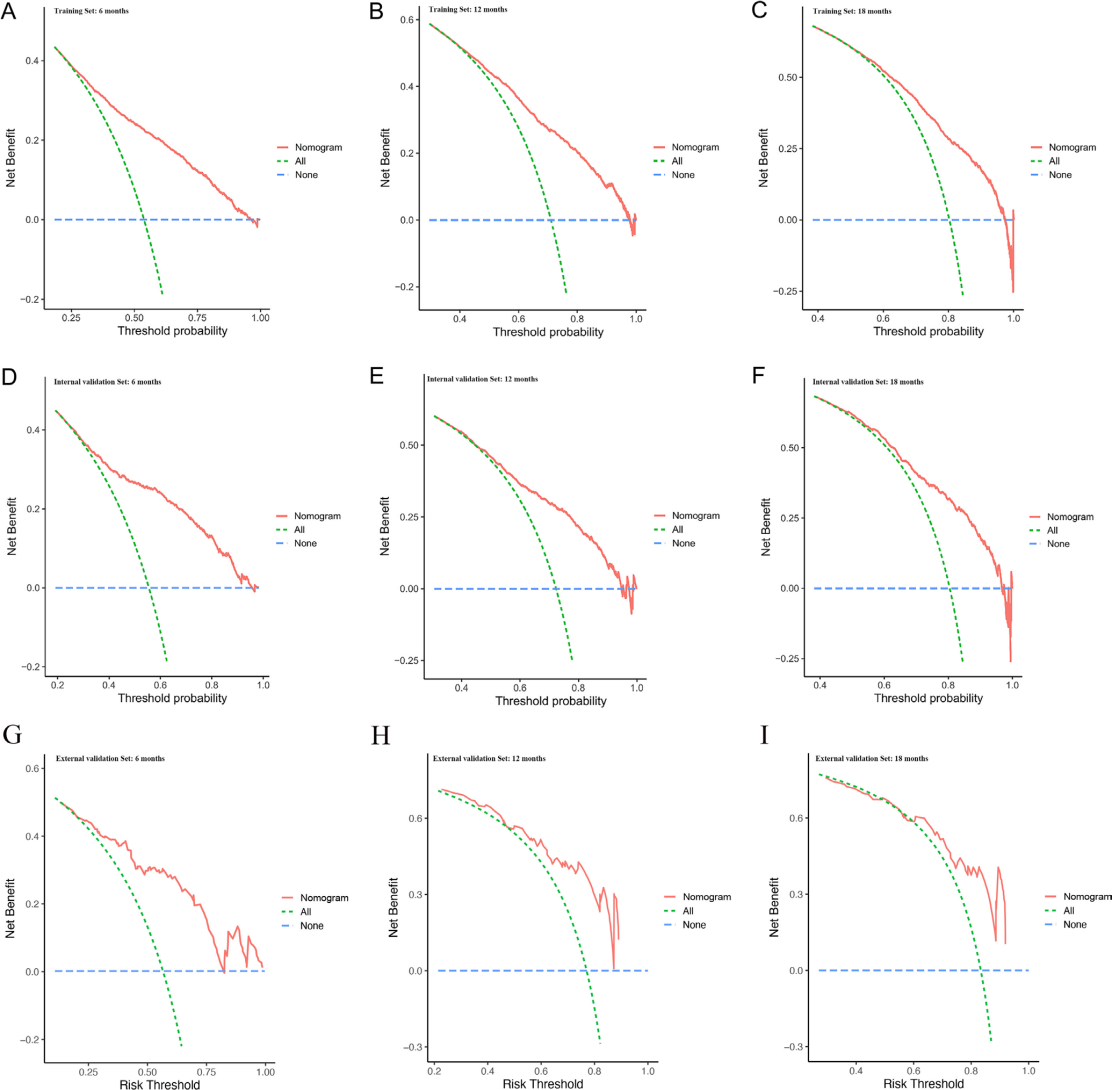
Decision curves for predicting the overall survival of patients at 6, 12, and 18 months. Training set (A, B, C); internal validation set (D, E, F); external validation set (G, H, I).

A nomogram was developed in the training set to predict the OS at 6, 12, and 18 months for lung adenocarcinoma patients with bone metastasis using the selected independent prognostic factors (Figure 3). Moreover, we developed a simple and user‐friendly web‐based dynamic nomogram (https://lzm‐qdu.shinyapps.io/my_DN/) based on our established nomogram. The concordance indices (C‐indices) for the training, internal validation, and external validation sets were calculated to be 0.703 (95% CI: 0.663–0.742), 0.731 (95% CI: 0.659–0.803), and 0.863 (95% CI: 0.754–0.988), respectively. The C‐indices for the training and validation sets exceeded 0.7, suggesting that the nomogram exhibited a satisfactory predictive capability. In addition, ROC curves were generated for the training and validation sets, and the AUC values were determined (Figure 4). The AUC values for the training set at 6, 12, and 18 months were 0.788, 0.767, and 0.756, respectively. Meanwhile, the AUC values for the internal validation set at 6, 12, and 18 months were 0.796, 0.757, and 0.756, respectively. Finally, the AUC values for the external validation set at 6, 12, and 18 months were 0.841, 0.819, and 0.815, respectively. The AUC values of the developed nomogram exceeded those of other independent prognostic factors, including T stage, N stage, and tumor grade. To assess the agreement between the survival probabilities predicted by the nomogram and the actual observed survival rates, calibration curves were generated (Figure 5). The calibration curves were found to be closely aligned with the 45° line, indicating a high degree of consistency between the predicted and actual observed survival rates. To assess the nomogram's clinical applicability, decision curves were created (Figure 6). The decision curves revealed a larger threshold probability range for the nomogram, indicating that the nomogram significantly increased the clinical benefit.

### 3.4 Risk Classification System

**FIGURE 7:**
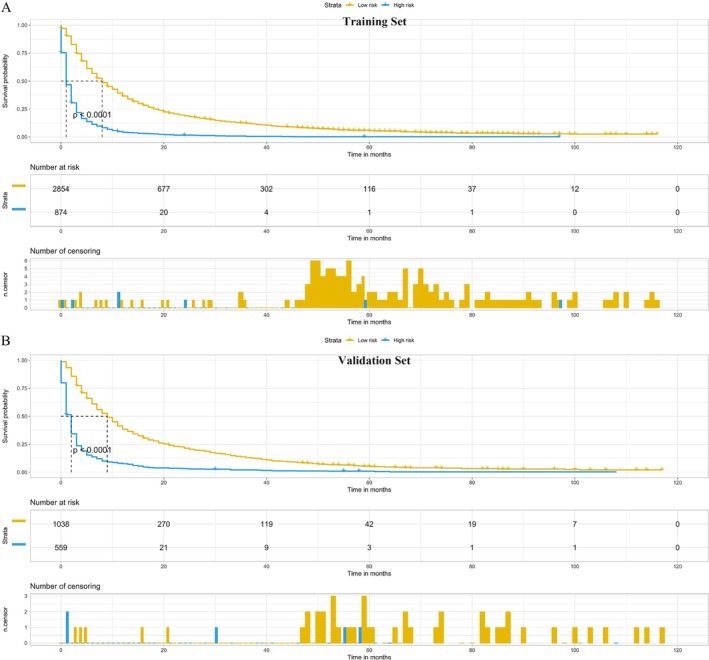
Kaplan–Meier (KM) survival curves. Training set (A), validation set (B). *p*‐Values < 0.05 were considered statistically significant.

The patients in the training and validation sets were categorized into high‐risk and low‐risk groups based on their total scores from the nomogram, and KM survival curves were constructed. As shown in Figure 7, the KM curves revealed that patients in the high‐risk group had poorer prognosis compared with those in the low‐risk group. In the training set, the median survival time was 1 month for the high‐risk group and 8 months for the low‐risk group. In the validation set, the median survival time was 2 months for the high‐risk group and 9 months for the low‐risk group.

### 3.5 Prognosis of Patients With Different Metastatic Sites

**FIGURE 8:**
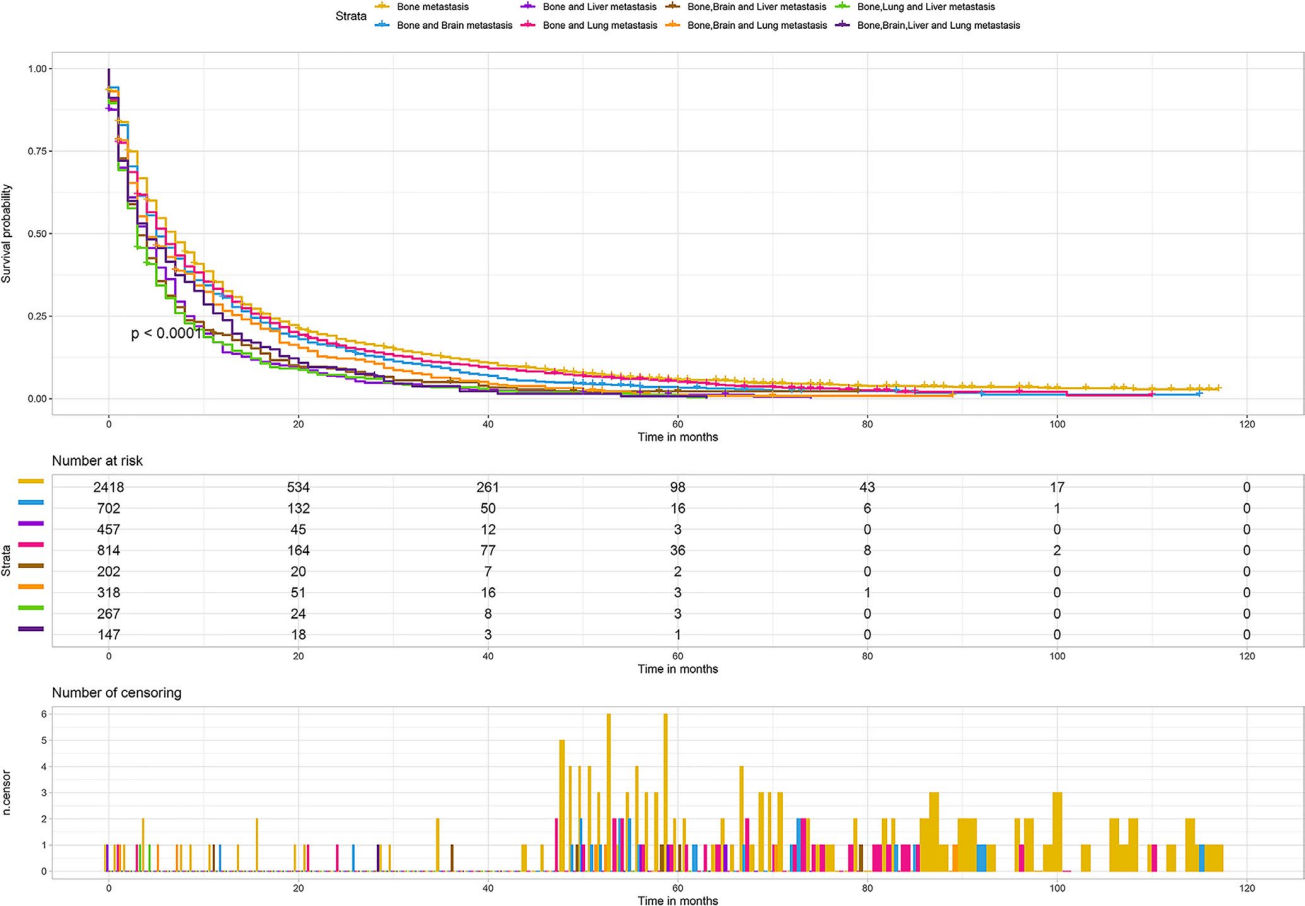
Kaplan–Meier (KM) survival curves were drawn according to different metastasis sites. KM survival curves of lung adenocarcinoma patients with bone metastasis and other different metastasis sites.

**FIGURE 9:**
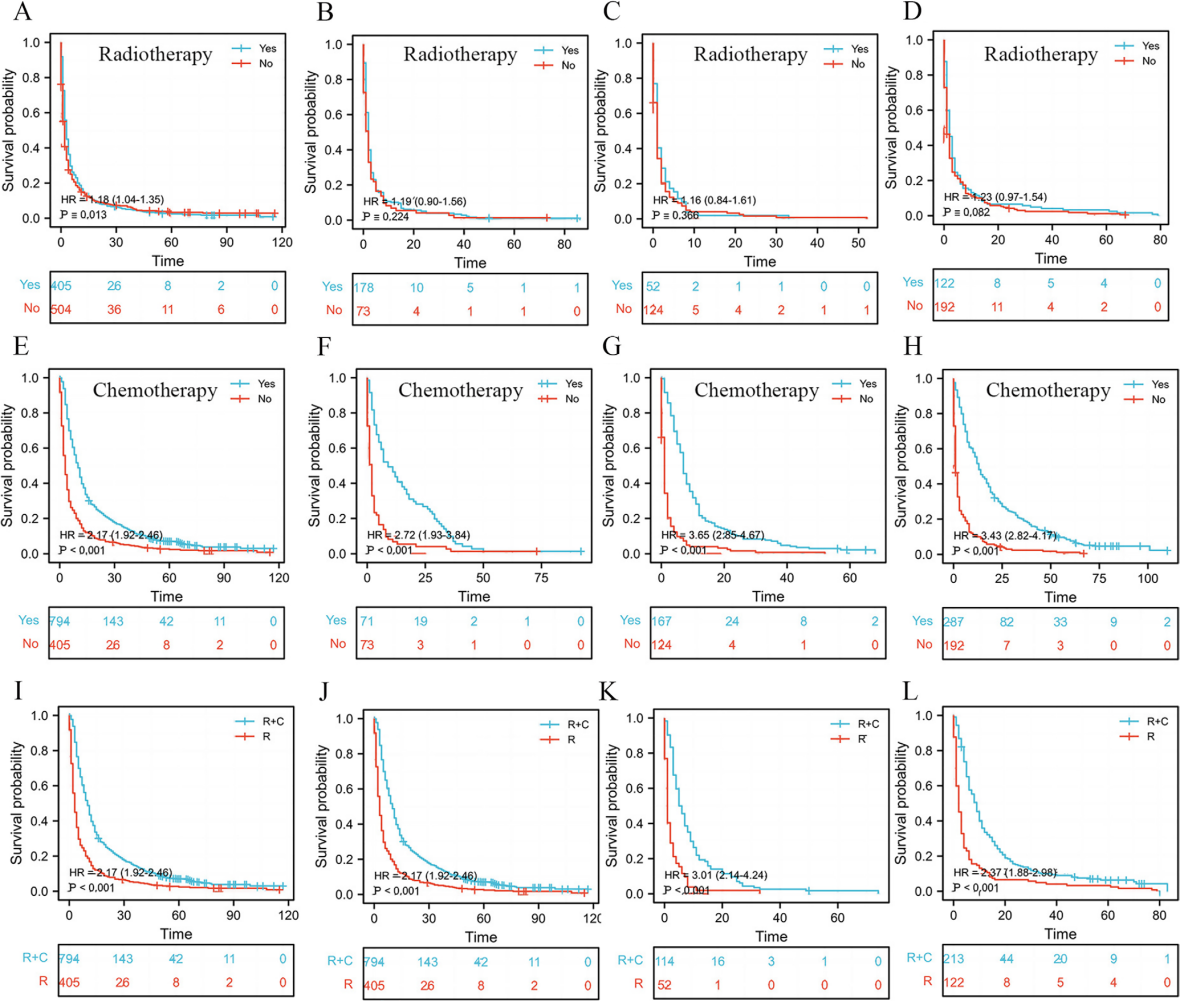
Kaplan–Meier survival curves of patients with different metastasis sites receiving radiotherapy, chemotherapy, and combined treatment. Subgroups of bone metastasis (A, E, I), subgroups of bone and brain metastasis (B, F, J), subgroups of bone and liver metastasis (C, G, K), and subgroups of bone and lung metastasis (D, H, L). R, radiotherapy; C, chemotherapy; *R* + C, combined radiotherapy and chemotherapy. *p*‐Values < 0.05 were considered statistically significant.

To assess the impact of different metastatic sites on patient prognosis, we created KM curves. As illustrated in Figure 8, patients with bone metastasis alone had the longest median survival time (7 months). By contrast, patients with bone and liver metastases had the shortest median survival time (4 months). The median survival times for patients with bone and lung metastases and those with bone and brain metastases were 6 and 5 months, respectively. Based on the nomogram, the total score of chemotherapy was higher than that of radiotherapy, indicating that chemotherapy had a better therapeutic effect. To verify this finding, we performed the following analysis. We plotted the KM curves of patients in the four subgroups to compare the effects of radiotherapy, chemotherapy, and combined treatment. As shown in Figure 9A–D, the HRs of patients who received radiotherapy were all close to 1 compared with those of patients who did not receive radiotherapy. Almost all groups had a *p*‐value greater than 0.05, indicating the unsatisfactory efficacy of radiotherapy. As shown in Figure 9E–H, the HRs of patients who received chemotherapy were greater than 2 or 3 compared with those of patients who did not receive chemotherapy, and the *p*‐values were less than 0.05, indicating that chemotherapy had better therapeutic efficacy than radiotherapy. As shown in Figure 9I–L, the HRs of patients who received radiotherapy and chemotherapy were greater than 2 compared with those of patients who received radiotherapy alone, and the *p*‐value was less than 0.05, suggesting that the combination of radiotherapy and chemotherapy had better therapeutic effects than radiotherapy alone.

## 4 Discussion

The nomogram developed in this study specifically focused on lung adenocarcinoma patients with bone metastasis, offering greater targeting. In addition, risk stratification analysis was conducted, and a user‐friendly web‐based interface was developed to provide more precise and convenient guidance for clinical treatment decisions. Compared with previous nomograms, the present nomogram had a higher AUC value, included a larger number of patients, covered a broader age range, and incorporated information on radiotherapy and chemotherapy status and different metastatic sites.

Jatoi et al. [13] have found that married patients with nonsmall cell lung cancer (NSCLC) have longer survival and better quality of life. Specifically, divorced patients face more severe financial problems, and married patients have better mental states. The nomograms established by Fan et al. [14] and Yin et al. [15] for predicting the survival of lung cancer patients with bone metastasis identified independent risk factors, including age, gender, radiotherapy, chemotherapy, brain metastasis, liver metastasis, and marital status, which are highly consistent with the independent risk factors analyzed in the present study.

Previous studies have investigated the prognostic influence of chemotherapy drugs, including erlotinib, gefitinib [16] and epidermal growth factor receptor tyrosine kinase inhibitors (EGFR‐TKI) [17, 18], on NSCLC using nomograms. The results revealed that chemotherapy is an independent prognostic factor for all subgroups. Similarly, the nomogram developed by Shi et al. [19] highlighted that chemotherapy exerted the most significant influence on the prognosis of NSCLC patients with bone metastases. These findings are consistent with those of our study. As observed from the nomogram, chemotherapy is a significant prognostic factor for lung adenocarcinoma patients with bone metastasis.

In addition, the *p*‐values of radiotherapy for lung adenocarcinoma patients with different metastatic sites were all greater than 0.05, which indicated that radiotherapy had little effect on OS [20]. This view is consistent with that of the present study. However, radiotherapy can be used as a palliative treatment to alleviate complications (e.g., pain) in patients [21].

The liver's immunosuppressive environment hinders the immune cell clearance of tumor cells, facilitating liver metastasis [22]. Liver metastasis can impair the absorption and metabolism of chemotherapy drugs, thereby reducing treatment efficacy [23]. Lung cancer patients with liver metastasis show low chemotherapy sensitivity, possibly due to liver dysfunction [24]. Moreover, poor treatment response is associated with reduced CD8+ T cells and PD‐L1 expression at the edges of metastatic lesions, along with impaired T cell activation in the liver, which undermines the anti‐tumor immune response [25]. High levels of tumor‐associated macrophages in the liver suppress T cell activity, promoting tumor growth and metastasis [26].

Our analysis revealed that outcomes can vary depending on the tumor metastasis sites. Patients with lung adenocarcinoma who developed bone and liver metastases had the poorest prognosis, with a median survival time of 4 months. In a study involving 17 431 patients with lung cancer, those with liver metastases exhibited the worst survival rates [23]. Similarly, compared with bone and brain metastases, liver metastasis was the strongest factor affecting the poor prognosis of patients with lung adenocarcinoma [27].

According to Zhang and co‐workers [28], tumor size emerged as a significant independent predictor of NSCLC outcomes, with larger tumors being correlated with reduced survival rates. This observation concurs with our findings, where patient prognosis deteriorated as the tumor size increased. The rationale for this correlation may be explained by the increased difficulty in the surgical removal of larger tumors, which is often accompanied by a higher likelihood of local recurrence [28]. Furthermore, tumor size, along with lymph node involvement [29, 30] and distant metastasis [31], jointly affects the prognosis.

According to a Norwegian personal registry and census, the all‐cause mortality of unmarried patients (including divorced, widowed, and never married) with cancer was more than 15% higher than that of same‐sex married people without cancer [32]. The reason was that married patients had more social support, better living habits, a higher proportion of insurance, and a lower risk of depression than unmarried patients [33].

Our study revealed that advanced age is a critical factor contributing to poor prognosis in patients with lung adenocarcinoma. This finding is in agreement with the perspective presented in the nomogram developed by Shi et al. [19]. The reason for this may be that elderly patients have more comorbidities and have poorer tolerance to surgery, radiotherapy, and chemotherapy [34].

A meta‐analysis of 1365 patients with NSCLC showed that women had better prognosis and a longer survival time than men [35]. This could be attributed to women having lower levels of p‐glycoprotein, which extends the half‐life of vinca alkaloids, doxorubicin, etoposide, and docetaxel [25] and also results in a heightened sensitivity to these chemotherapeutic agents [26]. Genetic susceptibility is also related to better prognosis in female patients with lung cancer, with males exhibiting higher overall incidence and mortality rates in lung cancer than females [36]. Moreover, a previous study found that female patients with lung cancer have a longer median survival time than male patients because females have a higher likelihood of being nonsmokers and a lower likelihood of passive smoking than males [31].

The present study has limitations that should be acknowledged, including the absence of detailed radiotherapy and chemotherapy data in the SEER database, the lack of an intermediate‐risk group for precision in treatment options, and the absence of biochemical markers for comprehensive evaluation. The nomogram requires further optimization by more researchers.

Moreover, the calibration plot exhibits bias at the extremes, showing that the predicted survival probability for patients at extremely high risk is slightly lower than the actual observed survival probability. The reasons for this bias can be summarized as follows: the model is trained based on existing data, which may not fully capture all factors affecting prognosis, leading to prediction bias, especially in extreme cases, such as patients with very high or very low risk. Moreover, data in extreme situations may be relatively scarce, which will also affect the stability and accuracy of the model. To address the calibration plot bias in extreme cases, it is recommended to collect as much data as possible, especially for extreme situations, to improve the stability and accuracy of the model.

This bias may lead doctors to be overly pessimistic about a patient's prognosis, thereby influencing treatment decisions. For example, doctors might forgo treatment or refrain from attempting more aggressive treatment options. Doctors should be aware of this bias and use the model's predictions with caution, particularly for patients at high and low risk.

## 5 Conclusions

This study established a new nomogram for predicting OS in patients with lung adenocarcinoma and bone metastasis, and the validation methods confirmed the good identification and prediction ability of the nomogram. KM curves of risk classification and different metastatic sites can provide guidance for clinicians to treat patients. In addition, this study found that liver metastasis is the strongest factor that reduces the OS rate of patients, and chemotherapy is the most effective method for treating patients with different metastatic sites.

## Author Contributions

**Zhiming Liu:** data curation (lead), methodology (lead), software (lead), writing – original draft (lead), writing – review and editing (lead). **Min Zhang:** data curation (lead), formal analysis (supporting), investigation (supporting), resources (lead). **Shuo Han:** conceptualization (lead), methodology (supporting), resources (supporting), software (supporting). **Hao Zhang:** data curation (supporting), investigation (supporting), software (lead), validation (supporting). **Shengwei Meng:** project administration (supporting), resources (supporting), software (supporting). **Zhubin Shen:** data curation (supporting), resources (supporting). **Xuexiao Ma:** funding acquisition (lead), supervision (lead), writing – review and editing (lead).

## Conflicts of Interest

The authors declare no conflicts of interest.

## Data Availability

The publicly available data utilized in this paper are accessible through the SEER database (https://seer.cancer.gov/). Those datasets can be obtained by contacting the corresponding author.
